# Unconscious system-psychodynamics within a German 4IR engineering company in South Africa

**DOI:** 10.3389/fpsyg.2022.926245

**Published:** 2022-08-25

**Authors:** Claude-Hélène Mayer, Rudolf M. Oosthuizen

**Affiliations:** ^1^Department of Industrial Psychology and People Management, University of Johannesburg, Johannesburg, South Africa; ^2^Department of Industrial and Organisational Psychology, University of South Africa, Pretoria, South Africa

**Keywords:** system psychodynamics, CIBART-model, human-machine interaction, fourth industrial revolution, multinational company, South Africa

## Abstract

This article focuses on systems psychodynamics and particularly on the CIBART-model which explores conflict, identity, boundary, authority, role and task and how these phenomena work out on an unconscious level. Therefore, this article presents empirical findings on CIBART in a German multinational engineering organization operating in South Africa. For this study, 16 managers where interviewed on their transformation toward Industry 4.0 with special interest in the interconnection how CIBART aspects play out in the South African context. Findings show that all of the CIBART-phenomena are important during the transformation to more advanced technological levels and restructuring processes. Conclusions are drawn and recommendations are given how to deal with systems-psychodynamic influences within the organization.

## Introduction

Industry 4.0 workplaces are defined by rapid changes toward a higher degree of technology usage and often new technological developments, such as artificial intelligence, digitalization processes, algorithms and the increase of robots in the world of work (Chuang and Graham, 2018). Often these transformational and change processes in organizations are experienced as disruptive (Kruger, 2020). The new complexities of leadership and organizations are sometimes challenging to comprehend and manage. This is particularly the case due to the speedy changes in leadership and organizational dynamics and the increasing human-machine interaction (Oosthuizen, 2019).

Although organizational change has long been researched, the unconscious impact in change and transformational processes has been underestimated and under-researched (Durães et al., 2018). This seems to be particularly true with regard to multinational organizations which contemporary transform toward Industry 4.0 (Tan & Wu, 2017). A dearth of research focuses on technological transformation, however, the impact on human levels seems to be under-explored with regard to conscious and even more in terms of unconscious processes. This article aims at exploring the unconscious, systems psychodynamics in a selected German international engineering organization operating in the South African business context.

## The systems-psychodynamic perspective

Petriglieri and Petriglieri (2020) postulate that systems-psychodynamic scholarship focuses on the interaction between collective structures, norms, and practices in social systems and the cognitions, motivations, and emotions of members of those systems. It is well equipped to challenge arrangements that stifle individual and organizational development (Mayer, 2020). Systems-psychodynamic perspectives have thus become vibrant in psychological and interdisciplinary research settings. Individuals are described within system psychodynamics to deepen the understanding of the unconscious dynamics impacting on cognitive, symbolic, affective and behavioral processes within such systems (Cilliers and Mayer, 2019).

Systems psychodynamics allows for the study and interpretation of collective, interdependent unconscious and conscious individual, group and intergroup processes resulting from the interconnection between different groups and subgroups within a social system (Czander and Eisold, 2003). Systems psychodynamics provide us with tools to understand and create awareness about the conscious and unconscious psychodynamics operating in an organization. Using a consultancy system psychodynamic stance enables practitioners, researchers and others to work with conscious and unconscious dynamics, organizational structure and design and the interaction between the two (Amado, 1995; Tonelli, 2019).

The conceptual origins of the systems-psychodynamic perspective stem from classic psychoanalysis (Freud, 1921), group relations theory, and open systems theory (Miller, 1993; French and Vince, 1999). The systems-psychodynamic perspective is a formatively centered, psychoeducational process for the understanding of the unconscious motivations of behavior of individual and group dynamics within the system. One’s essential task is to thrust the boundaries of mindfulness to better understand the underlying meaning of organizational behavior, including the challenges of management and authority (Miller and Rice, 1976; Koortzen and Cilliers, 2002). Unconscious dynamics touch on topics, such as boundaries, roles and role configurations, structure, organizational plan, work culture, and group processes (Neumann et al., 1997). Researchers focus on the unconscious dynamics, centering it on relatedness, representation, and how authority is psychologically distributed, exercised.

Smit and Cilliers (2006) posit that the systems-psychodynamic stance studies the emotional task of the system, which may be filled with chaos, a lack of control, and difficult experiences such as competition, rivalry, jealousy, envy, hate, hostility, and aggression (Miller and Rice, 1976, Miller, 1993). As a result, leadership becomes difficult (in the event that it is not incomprehensible). Besides, connections and relatedness between subsystems as well as the control of these inside boundaries ended up being progressively complex. As a result, mistrust and distrust increase (showing the predominance of neurotic fear as well as a need of meaning and trust within the system). Since leaders appear to discover themselves de-authorized to arrange new roles inside their organizations straightforwardly, the system makes modern components as a defensive compensation for the misfortune of control (Huffington et al., 2004).

## The CIBART-model of systems psychodynamics

The interaction between the unconscious processes and organizational structural elements highlights the need to work with the CIBART-model. The CIBART-model is a six-dimension boundary model (Conflict, Identity, Boundaries, Authority, Role and Task) which is used to study and explore conflict dynamics. The model enables practitioners and researchers to study and diagnose the dynamics of intra- and interpersonal conflicts in and across groups and organizations (Cytrynbaum and Noumair, 2004; Cilliers and Koortzen, 2005).

*Conflict* emerges from the oblivious uneasiness characteristic within the work environment and employees’ presentation to the good and bad parts of the system (Cilliers and Koortzen, 2005; Koortzen and Cilliers, 2007). It too alludes to parts experienced inside the self, between the self and others, inside groups and between groups (Hunter, 2018). Conflict serves as a driving force in group execution, inventiveness, development and adapting to transformation (Mayer, 2008). It is anxiety-provoking, manifests intra- and interpersonally, and impacts leadership, role formation, conflict identity boundaries and specialist as oblivious designs of inner self resistances, such as fight or flight reactions (Koortzen and Oosthuizen, 2012). Typically, a result of the unconscious dynamics is to feel secure (Bion, 1961). The individual/group at that point tries to reduce uneasiness by matching with effective others and part from others to construct smaller, appearingly more secure, systems (Mayer et al., 2018).

*Identity* reflects the system’s uniqueness within the frame of its convictions, disposition, viewpoint or social and political points of view (Hayden and Molenkamp, 2004; Goldin, 2017). Identity alludes to the integration of the above—the system’s uniqueness through its mental characteristics (Campbell and Groenbaek, 2006). Typically, the nature of the leader’s role-behavior and the branding, climate and culture of the hierarchical system relates to identity (Oosthuizen and Mayer, 2019).

*Boundaries* are spaces of a physical and seriously psychological nature (Sher, 2010; Reciniello, 2014) over which trades take place in a system as well as a transitional or potential space filled with unconscious flow which exists inside and between groups, as well as the organization’s structure (Heracleous, 2004; James and Huffington, 2004). Boundaries are critical within the control of feelings. Boundary alludes to the line around and space between the individual’s and other employees’ parts in service of emotional assurance and control (Cilliers and Koortzen, 2005).

*Authority* as expressed by Obholzer (2001, p. 201) is the product of organization and structure, be it external, as within the organization’s sanction, or inside, as within the inner. This authority is utilized within the compelling completion of the essential task or shared tasks (Eisold, 2004) and in making official choices for self and others (Beck and Visholm, 2014). This authority can be “given from above” from management, or “from below” from subordinates, or “from within” the group (self-authorization) or from other groups. Authority in this way alludes to the right to perform the essential task as formally authorized by the system represented by leaders from above, colleagues from the side, subordinates from underneath and by workers themselves from inside (Hazarika et al., 2018).

*Role* alludes to the depiction of what ought to be exhausted to perform with respects to obligations and errands inside a particular boundary (Hayden and Molenkamp, 2004; Cilliers and Koortzen, 2005). Usually, managers have obligations and tasks to fulfill and therefore take on certain roles which can be formal (e.g., a manager’s role) or informal roles (e.g., the critical one, the crazy one, etc.), self-ascribed or ascribed by others. Authority is tied to positions or roles (Beck and Visholm, 2014). Three sorts of roles are recognized, specifically (1) the normative role referring to the job description and content; (2) the existential role, referring to how the team believes it is performing; and (3) the phenomenal role, which relates to what can be inferred by other’s mostly unconscious behavior toward the team. Incongruence between these distinctive roles causes anxiety and destitute execution (Steyn and Cilliers, 2016). Role alludes to the boundary around a set of errands, obligations and duties on a particular level of authority and shows as the standardizing (the cognizant and unequivocal substance), the existential (the employee’s introjected past encounters and identity characteristics such as values and inclinations) and the phenomenal part (the projections that the representative gets from noteworthy others within the hierarchical framework; Czander, 1993; Obholzer and Roberts, 1994; Cytrynbaum and Noumair, 2004).

*Task* is the essential building piece of work (Cilliers and Koortzen, 2005). Employees may be included in essential and auxiliary assignment functioning as well as off-task and anti-task conduct (Obholzer and Roberts, 1994; Cytrynbaum and Noumair, 2004). The organizations can be a multi-task system, for example the university has three essential tasks, *viz.* teaching students, creating research publications and giving pertinent community service (Rice, 1970; Rogers, 1976). According to Lawrence (1985, p. 235), the (essential) task may be an instrument for request to understand the substances of the association and other social courses of action of (the workforce).

## Research methods

### Research paradigm and design

This empirical study is based on a qualitative research paradigm (O'Cathain, 2020). The research design was developed based on a collective case study design (Wilson and MacLean, 2011), in which selected professionals within the South African branch of a multinational German-owned Engineering organization were invited to participate in interviews. The paradigm, design and approach were chosen to contribute to an in-depth discourse on unconscious dynamics within the South African organizational contexts.

This new in-depth knowledge is necessary to explore the impacts on managers’ unconscious experiences and levels of CIBART and the systems surrounding it. Findings can be used to create awareness within organizations and to adjust guidelines, policies and development plans during organizational change processes.

### Organizational context and sample

The organization is a multinational Engineering company with a subsidy in Johannesburg, South Africa. The company is specialized in high-tech water engineering and one of the leading European companies in this field.

The sample consisted of the total of 14 South African and one German manager working I the organization at the time of the interview. The interviews were conducted in English with managers in middle and top management positions. The total of the managers consisted of 13 South African (11 white—English and Afrikaans-speaking—male managers, one German male and one Colored South African female manager). At the time of the interview, the participants had spent between two and 23 years working in the organization. The age of the managers ranged between 32 and 60 years. All managers held a university degree or diploma. Inclusion criteria for the participants were: holding a managerial position in the organization researched. The researchers aimed to include all managers working in the subsidy and managed to do so. Both researchers conducted the interviews within the organization.

### Entry to the field and organization and ethics

As an external consultant to the company one researcher was invited to conduct the research for training and development purposes. All participants were informed about this and ethical considerations were applied.

Ethical considerations and qualitative quality criteria were followed, as described by Clarke and Hoggett (2009) to conduct the research study by adhering to ethical considerations, such as informed consent, privacy, confidentiality, respecting of rights, transparency and not causing any harm. Ethical approval was provided from an academic body, a German university, as well as in terms of organizational consent by the organization and the individual participants. Participants provided consent and attended voluntarily.

### Data collection, analysis and reporting

Data were collected through 30–45 min semi-structured interviews. The topic of the interviews was “Managing the changes of the Fourth Industrial Revolution successfully.” Data were collected (recorded and transcribed verbatim) and analyzed by the two researchers. The purpose of the interviews was to study participants’ thoughts, feelings and experiences in the organization, transforming toward Industry 4.0. Sample questions include: what is the 4IR for you? How do you experience it? How do you experience the dynamics within your team? What experience do you make working in a dynamic team, transforming the organization? What are your roles, what are your tasks within the organization?

Data analysis was conducted through thematic analysis, which included the following steps: (1) reducing the text, (2) understanding it as a subjective experience of individuals; (3) interpreting it on the basis of the theoretical background of systems psychodynamics (Clarke and Hoggett, 2009). As according to Obholzer and Roberts (1994), data were analyzed in terms of normative, existential and phenomenal roles, while referring to the hermeneutic circle (Alvesson and Sködberg, 2010). Data analysis was informed by the unit of analysis. The 16 participants formed the collective group of individuals. According to the systems-psychodynamic processes, the individual acts on behalf of and as a representational member of the group (Cytrynbaum and Noumair, 2004). The micro-level thereby reflects and represents the macro-level of the organization.

Findings are reported in a qualitative reporting style. Data are reported based on a double-bind strategy, namely to gain a deeper understanding through intrinsic insights and to fulfill an instrumental purpose, that means providing information to the organization (Denzin and Lincoln, 2005). In the findings section, findings are reported according to the CIBART-model themes, whereby data were analyzed in depth, aiming at a complex understanding of the data in relation to the content of the individual participants as part of the collective case study group.

### Quality criteria

Employees shared wealthy subjective experiences which led to meticulousness within the quality of the data, its examination and interpretation (Johnson et al., 2020). The researchers utilized differing sources, procedures and speculative approaches to ensure legitimacy through triangulation by combining different theories and methodologies (Angell and Townsend, 2011). Conformability and transferability of data (Creswell, 2013; Pope and Mays, 2020) were progressed through intersubjective endorsement forms and the utilization of set up speculations and procedures which were built up among the researchers (Yin, 2014), while exhaustiveness was progressed through thick delineations and clear forms. The study further gives an in-depth understanding into the revelations and topics, but does not allow generalizability (Lincoln and Guba, 1985; Creswell, 2013).

### Limitations of the study

Because the literature on transforming into Industry 4.0 organizations is very limited when it comes to the analysis of systems psychodynamics and unconscious processes, the discussion in terms of research with regard to this specific aspect is also limited. Further, the study is limited as a collective case study within a single organization. This study is strongly limited to its specific context and cannot be generalized. It can, however, be viewed as a rigorous insight into a specific context and can therefore be a starting point for other studies aiming at focusing on unconscious dynamics in times of transformation toward Industry 4.0.

## Findings

The findings show how system-psychodynamic processes, as described in CIBART impact on the leadership within the Engineering organization which is operating internationally and which is striving to transform toward a leading 4IR company. The article presents and discusses the findings with regard to the relevant contemporary literature on system psychodynamics and CIBART and draws conclusions with regard to managing leadership and change toward 4IR transforming companies. Based on thematic analysis, the following will provide the findings with regard to the CIBART-model. Selected direct quotes from interviews will be presented to give the reader an idea of what the direct quotations reveal.

### Conflict

Managers do not talk about conflict directly, but when the researchers analyzed the data, implicit conflict appeared in the dataset as underlying issues. The conflicts are experienced on different levels: on the societal, the organizational, inter- and intrapersonal levels.

#### Societal conflict level

Managers point out that they find themselves in a conflict having and wanting to promote 4IR on all professional levels, however they struggle with the societal infrastructure to really become part of the global 4IR movements. Hence, the majority of managers highlight that they would like to implement 4IR systems (and have done so partly), but that the basic infrastructure on societal levels is hardly in place. For example, there is a lack of speedy internet (e.g., P12, P13), often there are electricity and water shortages and the IT infrastructure and hardware are not available (e.g., P10, P13). Often, South African infrastructure struggles with building competitive networks which can be used to compete on global levels (P8, P10, P12, P13), and politics which supports the technological-economic advancements (e.g., P5, P15). P12 highlights:

One challenge that always comes to mind is … our system needs to work. Ja. Bad lines, … So I think that the downside always is that you need a stable line or whatever you want to call it – infrastructure.

Further, in society there are two clashing trends, namely one trend which aims at belonging to the global 4IR community and which is supported by 13 out of 16 managers, while 3 others highlight that African countries need to look after themselves, need to slow down in speed of change (P12) and develop own tools which reflect the African realities and not the ones of the industrialized countries (P13). P13 is in favor of creating new technologized tools to monitor customers and products in Southern Africa while saving investments for the company.

For me, it’s basically how are we going to do things in the future with regards to new technology, It’s a more automated, more AI and virtual reality driven. Not this face to face. I think it’s going to be a lot more screen to screen. You never know when the next breakdown call is going to be, so I see our opportunities will be different than from a production point of view. I think virtual reality is something that we will get into because I’m responsible for the whole sub-Saharan Africa. To travel to a country for one day to go check something is expensive. And it takes you three, four days to get there. It’s costly. So if we can equip ourselves with something that we can see what’s happening on the other side, it will be a great benefit. I think a service tool that we will be able to use because your reaction time is quicker, your responses are quicker.

While on the societal conflict level strong challenges and conflicts are based on the missing infrastructure for the implementation of 4IR technological advances, managers are conflicted between their wish to advance and play on the global 4IR level and being confronted with the realities of developing countries which find themselves in a recession.

#### Organizational conflict level

On the organizational level, managers highlight two conflictual issues, namely on the one hand having to manage the intra-organizational communication between the different managerial levels and the employees and on the other hand having to manage their relationship with headquarters which are based in Germany.

The entire sample of managers interviewed highlights that communication is a challenge within the organization. They feel that underlying conflict occurs due to the fact that employees build up fears about the rapid changes and potential job losses and with regard to the future of the organization. Managers mention that this missing communication with the employees on all levels leads to conflict within the organization, given that there is a total lack of information. P1 explains how conflict can be addressed and avoided within the organization:

There should be some sort of communication with bulletin boards or newsletters or something just to show we are moving in terms of industry. So other employees, like store men, pump attendants, they can see this and not get a shock. Oh, no, we will be replaced eventually by robots.

Conflict emerges when employees experience insecurities, when they are uninformed and when their fears and ideas are taking over their knowledge about the processes. The second aspect of organizational context is experienced in the tension between international and local decision-making. Managers feel that the German headquarters are making decisions which might not be context relevant and which might be to the disadvantage of the subsidiary due to not comprehending the complexity of the African context (e.g., P2, P3, P6, P15). P15 explains:

From a business perspective, we are going that way. We know we’re going to Ariba; the system …whoever is on Ariba and … whenever that person wants something you will automatically be fed that inquiry … it’s probably going to be brilliant, but at the same time in a country like South Africa, I don’t think that that’s the answer to our problems. … we going the creditors’ way where our creditors are going to Manila, and is going to be paid from Manila., the payments are being processed in Manila. And I have a problem with it in that, okay, my debtor staff are safe, my creditor staff are not. Now the problem this is as a South African, as a person living in South Africa, do we not see that we have a labour problem already, that we have an unemployment problem already?

Conflict in the organization occurs when jobs are being lost due to restructuring processes and decentralized, computerized programs. Organizational conflict exists in the tension of internal communication which is experienced as missing or incomplete and with regard to a lack of trust by the German headquarters with regard to the decision-making of the subsidiary in South Africa. This leads to conflict on the South African managerial side in that they feel that managers have the relevant knowledge to make context-specific decisions which are adequate while the German management does not have the context-specific knowledge but wants to control the South African subsidiary and make inadequate decisions on their behalf.

#### Inter-psychological level

Managers point out that there is implicit conflict within the organization on interpersonal levels. On the one hand, managers highlight that due to the 4IR there are power shifts which need to be accepted and dealt with. These power shifts advance the individuals who hold knowledge with regard to latest technological trends in their professional area. P2 comments:

With the new technologies, there are powershifts which are occurring. The ones who hold the power will be part of the global 4IR and they will be competitive. The ones who can do programming and manage the new technologies. But the ones who do ordinary jobs will be replaced by machines. The shift in power will only be good for the ones who hold knowledge already. This will create more problems.

On the other hand, managers highlight that they are conflicted between aiming to advance and speed up to keep up with the global trend of the 4IR and at the same time having to look after employees which are not in a situation in which they can progress to the next level, considering they have—as a consequence of the Post-Apartheid era and misleading politics—not yet reached a basic standard of education, skills and abilities to keep up with the current work challenges (e.g., P6, P15). P6 points out:

How will the people who are unaware of what is going on become part of this 4IR? There are too many people here who cannot yet progress to the next level since they lack knowledge, skills, education, …all one needs. It will make the divide bigger.

On an interpersonal level, several managers anticipate conflicts with regard to the radical changes and the split between people who turn to the new ways of living and working and the people who will be left behind.

#### Intrapersonal level

On an intrapsychological level, managers refer to four aspects. They highlight that they are in an inner conflict with themselves with regard to having to cope with local and global pressures, having to apply new technological innovation and be professional with a human touch and at the same time, cope with a variety of emotions and with the experience of their personal limitations (e.g., P8, P9, P11, P12, P14).

Firstly, managers feel conflict about having to cope with local and global pressures. Secondly, they aim to learn new professionalism with technological aspects, but also retain the human touch. Thirdly, they are in conflict with a broad spectrum of emotions. Finally, they have to accept and manage their personal limitations. P3, for example, points out that to manage business interactions and conflictual situations, the human touch is needed, as well as intuition and a gut feeling to analyze the situation and make decisions:

I can see certain changes towards automatization in departments, especially in sales, customer consulting, but I think to certain people, especially on our side, you’re going to need that human interaction. That human touch that sort of, I know, yes, you can go on history and you can go on your numbers. But when it comes to that gut feel, we need to be able to look at it, you know.

**Figure 1:**
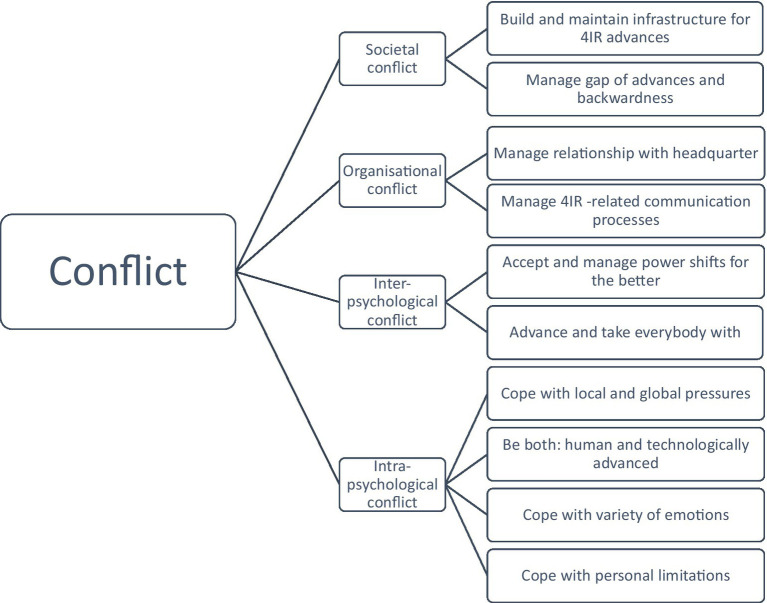
Conflict.

The excerpt shows clearly the conflict of the inner question (Figure 1) how to be a 4IR transformed individual while at the same time staying “human.” This is a question that appears to be conflictual for many managers.

With regard to conflicts experienced, it appears to be highly conflictual for managers to deal with their own, intrapsychological aspects, because most managers refer to intrapsychological conflict with regard to different issues. This shows that managers need to build up knowledge and insight into the 4IR processes to understand them, manage them and construct and gain new knowledge. The main underlying conflict seems to be a split in the mindset which is build up around the issues how to manage the 4IR at its best for the employees and the organization while aiming at closing the gap between the global and local context.

### Identity

During the interviews, the managers showed that the question of their identity has become relevant. Several of the managers highlight that an important aspect of their professional work is based on a personal and humane contact with their colleagues and customers. This personal contact as a human professional builds the basis of their identity. They are professionals who care for the employees (P4, P7, P8, P16), broaden their horizon in the context of working with others (P8, P11, P12, P16), deliver quick solutions ((P10, P14, P16) and care for the environment and communities (e.g., P4, P8, P9). Their identity is strongly built around human contact and care. P7 highlights that his work identity is a great deal about caring for the customer and about being human.

The systems get more automated in order to make the work more efficient. But … you wouldn’t be able to take away the human component. In my field of expertise – it is based on intuition and different practices of knowledge. For instance, I cannot tell a computer system to accurately gauge or anticipate what a customer might do. You may program for these types of things, but getting a feel for the customer is not something that a machine will probably accurately do.

At the moment, managers feel that there is a shift in identity toward becoming a human-technology-orientated and -driven professional. That means that managers know how to balance out their identity in terms of their self-definition with regard to becoming a human-technology-mediated professional who needs to balance human-machine interactions and find his/her personal standpoint within the new system elements (P1, P3, P4, P5, P7, P10, P15).

The integration of technology is not only seen as being a work tool, but also as technology becoming part of their identity and part of responding to the question of “who they are,” since managers have integrated technology already largely into their professional organizational work, but also into their private lives. Several managers also highlight that part of their identity is being a technology-informed individual who presents new solutions to the organization for optimization of organizational and technological processes (P1, P4, P5, P7).

Managers further feel that they have to develop an identity which needs to adhere to the attributes of being innovative (P1, P3, P4, P5, P7, P9, P10, P13, P16), solution-finding (P5, P7, P8, P9), analytic (P5, P14, P15), and intuitive (P7). P4 explains his profession and mentions that technology has become a part of himself, his life, thinking and living:

Technology is a part of me and my work. Many years ago I have already worked in the forefront of technology. It is part of my work life and part of my life at home. We implement and use smart systems all over.

Further, it becomes a very important aspect of the managerial identity of being of African origin, working and living in a South African context. The continental and national context of the identity is becoming very important with regard to the managerial 4IR identity since the contexts defines a demarcation of managers’ identity in terms of advantages and disadvantages and specific contextual challenges. While resonating with European and global 4IR mindsets, managers are also resonating with the limitations within their African living and working contexts—and their specifics. P5 explains:

But a company is just, it’s not just machines. It’s the people in the company. It’s the personal touch that a customer has with a person. Not a program. Most people I know, that, they will rather go sit and talk to a person than call them. And now you’re telling them no just fill in a form on the internet. There. you’ve got your pump.

**Figure 2:**
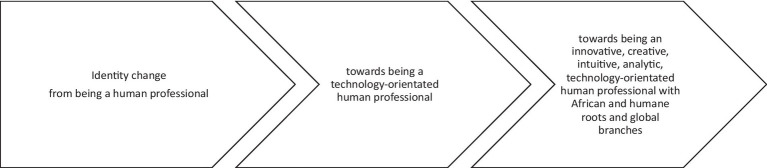
Identity.

Identity is very much connected to building personal contact and work identity and relationships are built strongly on personal contact and work is therefore built strongly around a personalized work identity. Generally, there is a shift in identity construction from a rather narrow identity definition toward a more complex identity definition which integrates technological aspects on the one hand and attributes and characteristics which have been defined as valuable in a 4IR setting on the other. Identity is clearly defined with a contextual relation of managers toward global and local, Pan-African and South African contexts. An overview is given in Figure 2.

### Boundary

In the 4IR setting, managers define clear boundaries. These boundaries are seen in the context of generation and age boundaries, educational and skill boundaries, language barriers, geo-economical boundaries and emotional boundaries. The majority of managers refer to the idea that the 4IR creates a boundary between “the old and the young generation in the company” (P4, P9, P12, P13, P14, P15, P16). The boundary is defined by the specialization of knowledge according to age. Consequently, the older generation is described as experienced, knowledgeable, informed and skilled, the younger generation is described as technologically affine, technologically knowledgeable, and interested. The age boundary is not set in finer detail, only “older and younger generation.” P9 points out:

The people should have the possibility to share their ideas…if you look at the new generation, which has grown up with digitalization… they have a different approach of their life…they have decent opinion and they don’t like the style of old companies how they are steered, …they’re looking more, from my perspective, to environmental issues. Ja, they’re looking for green. And if they’re getting aware, these companies that don’t, doesn’t care, they step out, they go to a different one. They want to change the world to a better world and they want participating companies who are on that way. People have to share ideas and the companies have to have a mission. For a better world. And then they will work and then the people will participate and they will make the change.

For this manager, the new mission of companies is to go green, be participative, work for a better world and transform. For him, 4IR is not only about technology, but also about “going green” and being responsible. The boundary is drawn between the older and the younger generations in the context of being responsible leaders with technology versus acting less responsible.

An additional boundary is described in terms of the employees with skills and education on the one hand and the ones without skills and education on the other hand (P3, P5, P7, P8, P12, P15). The knowledgeable group is described as profiting from the situation of transforming into 4IR, being excited and driven by new challenges. The second group is described as rather unknowledgeable, uninformed and disturbed by the circumstances and the changes. P16 emphasizes:

These days everybody gets a computer and there’s a program in the computer, and everybody just enters data and out comes the result. But they do not necessarily understand what the computer is doing, there’s no background. Maybe I was fortunate enough to live through an age where you had to do it manually. And then now, no one, in my opinion, thinks anymore because I have a computer program to do it for me. It splits up all the results, it does whatever I want – but you don’t know how it does it. So that’s what I find: people are lacking the basic knowledge because it’s not really necessary.

The manager differentiates clearly between the people who know the system and who have a deeper understanding of the system and programs and the other ones who have a superficial understanding on how to operate programs to a certain degree, but lack basic knowledge and understanding. Further boundaries are described in terms of language barriers and boundaries and the fact that not even Google translate can overcome the anticipated language boundaries which impact negatively on the communication among employees as well as with customers (P8, P10, P12, P14). In this sense, managers describe the failures of the 4IR technology implicitly, by showing that these boundaries of communication due to language barriers cannot even be overcome by technology. P8 explains:

I think we also have the language barrier where I read something – I’m Afrikaans, English is not my first language as I say – so we can read the exact same paragraph and this is how I interpret it, and you as a Zulu-, Xhosa-, Venda-speaking person read the exact same paragraph, and you interpret it completely differently. And I think that that’s one of our challenges – the language barrier.

P8 describes the boundaries caused by using different first languages, however, the manager later on also describes literacy “barriers” and that employees in the organization struggle to even fill in basic forms, for example their pension funds. Managers also point out that there are strong geo-economical boundaries which they see between the developed and the developing countries in terms of opportunities of 4IR applications and development and with regard to the boundary of the ones with education and the ones without (e.g., P7, P11, P14, P15, P16). The boundaries seem to be the demarcation of the transformed and the transforming, of the advantaged and the disadvantaged. P16 explains:

We can’t change the system for South Africa. And I say you do not change the system for South Africa, you are changing it for Africa. Because Africa does this, it’s not South Africa. Africa does it. I know I deal with it. Ja. Ja. It’s my baby. Now, before you even start training the people of South Africa, you have to train them globally, you have to bring in the knowledge to come and work here on how it is done here. And you’re trying to tell them that whatever they are telling you, does not apply here, and it will never apply here, and they are wasting their time trying to change the system in Africa.

**Figure 3:**
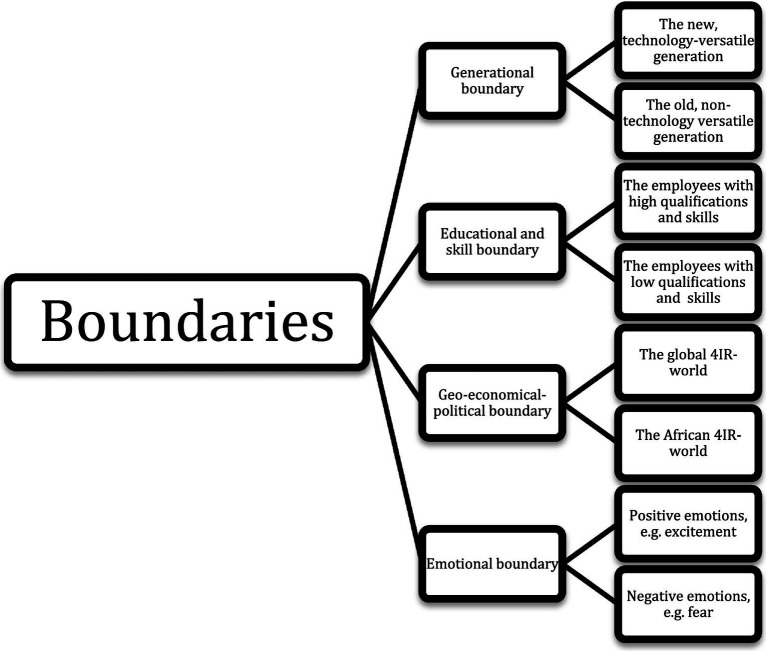
Boundary.

P16 is convinced that systems which are used globally do not apply to African contexts and that this draws a boundary between the world and Africa. Finally, boundaries exist between the ones who experience positive and the other ones who experience negative emotions. The 4IR world of work seems to be split by emotional boundaries which do divide the world of the winners and the losers of the 4IR based on their emotional experiences. Managers talk about the employees who fear the 4IR and others—and here they include mainly themselves as well—who are excited about the 4IR. So, a boundary is drawn between the ones with negative and the ones with positive emotions and mindsets regarding 4IR. This boundary seems to interlink with the more educated and skilled ones being excited (Figure 3), seeing their opportunities and the less skilled ones being more fearful, not being equipped to see what is coming.

The boundaries experienced in the organization are drawn along generational, cultural, racial, language, educational and emotional lines on the individual and organizational levels within the organization, as well as on a global level in terms of the advanced world and the headquarters and the unadvanced world and the subsidiary.

### Authority

In terms of authority, managers see a shift in authority in the period of transformation toward the 4IR which relates to certain attitudes, emotions and behaviors. Thy highlight that technology brings authority within the technological age, which they call the “new authority.” This new authority is based on new technological systems, such as smart systems, digitalization, robots and automation, 3DModelling etc. All managers refer to this new authority which is demanding its place in the field of work. P6 points out:

It is all about technology. Technology and the knowledge about it runs the world and our professional life.

A high number of managers highlight that employees in the organization need to turn toward a positive mindset regarding the 4IR with its new authority, to use it in its own right for the betterment of the work, including an increase in speed (P2, P3, P5, P8, P9, P10, P12, P15, P16), 24/7 work (P5, P12) and new systems (P1, P5, P7, P8, P9, P10, P12-P16). According to the managers, the new authority needs to be welcomed by applying four aspects: a positive mindset (e.g., P14), the ability to understand complexities of the new world of work (e.g., P3, P5, P7, P8, P11, P13, P15), to gain new advanced skills and apply them (all managers), and influence actively the new 4IR changes and control the new technologies toward the betterment of employees and the world (e.g., P8, P12, P14).

P5, for example, emphasizes that advanced skills need to build onto basic skills, that complexities need to be understood and that then, if applied correctly, the new systems and automation can support the advancement of the employees and the organization:

I do believe that, like anything in life, you need to know the basics before you can make an advanced system. So, if you personally don’t know the basics, how can you run an advanced system? So, I do think that people still need the skills to work from the basics. They need to understand the product they’re making and how to do it. Because then you can integrate it into the automation, make it faster. Because automation isn’t really there to replace the person, it’s more there to speed up the process.

**Figure 4:**
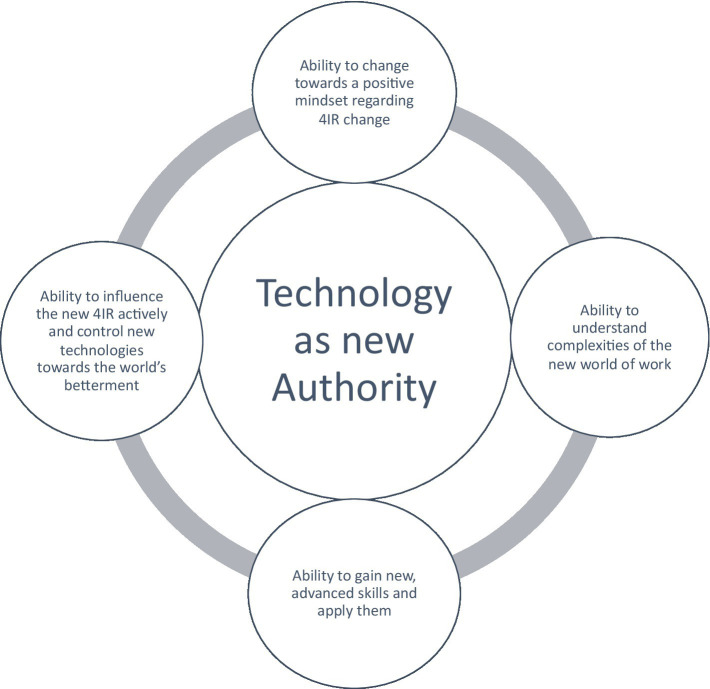
Authority.

This manager also points out that the 4IR technology and authority are not there to replace the employee (Figure 4), but rather to contribute to the speed of production. Authority must thereby be controlled by the humans as well as not just taken for granted and be followed.

The authority is given to the “new technology,” an abstract concept which is seen as taking over the authority in the world. It seems managers think that they need to keep up with the speed of the technological authority and silently adhere to it, try to please it by adjusting to the technological age, the needed skills and the new mindset. There seems to be an underlying perspective as if technology becomes a global, an organizational and individual authority which measures everything. It is as if technology is the new ruler of the organization.

### Role

During the 4IR, managers highlight that they need to take on new roles, such as the role of communicator, solution-finder, inventor, creator/maker and leader/guide. The most important role is the role of communicator (all managers), since managers feel that there is not enough communication within the organization which provides employees with information. Therefore, the role of communicator is not optimally fulfilled and needs to be addressed. P1 presents ideas regarding the roles that need to be taken on regarding communication and why:

There should be some sort of communication with a bulletin boards or newsletters or something just to show we are moving in terms of industry. So other employees, like store men, pump attendants, they can see this and not get a shock: “Oh, no, we will be replaced eventually by robots.”

The communicator role needs to be taken on by the leaders and managers of the organization and it is interlinked with the role of the leader and guide. None of the managers criticizes leadership and guidance directly, however, indirectly it is emphasized that the leadership roles of different unities or individuals do not necessarily match the needs of the context (German headquarters imply views on the African context, South Africa needs job creation not job losses, the organization needs to provide adequate training, leaders in the organization should listen to the innovative ideas of the managers etc.).

Additionally, several managers describe that the organization needs innovation, but only a few individuals take on the roles as innovators. Some do, but they are not heard and therefore they take their ideas out of the organization to sell the patent to someone else (e.g., P3, P4, P5, P6). Some also highlight being innovative regarding the 4IR.

We need to do our own research and develop…. me personally, I am a maker…I wouldn’t want to just hand my idea over. I think it’s an ownership thing. I’ve got this idea and I want it to go in a certain direction. I think a lot of people might think the same. I come up with an idea to you and I hand it over to you and you go – do your own thing…. I just think ownership. I want to see it succeed with the vision I’ve had. I don’t necessarily mind if we, but it’s also for the company. We can. I mean, I now wouldn’t be able, I wouldn’t be able to do it on my own. Your initial outlay is expensive. Ja. It’s not for me to make money, it’s to bring something into the industry.

This manager would like to establish a cooperation between the organization and himself where he can bring in his own idea and research and have the organization buy into the idea and help to produce the component to make the life of the farmers easier. To him, 4IR is about making innovation happen between managers and the organization to work for the greater good. Additionally, individuals emphasize that the roles of being a creator and maker are important to them, but there does not seem to be space for this role within the organization and that is why individuals usually are being creative in their leisure-time and have hobbies where they can exercise their creativity.

**Figure 5:**
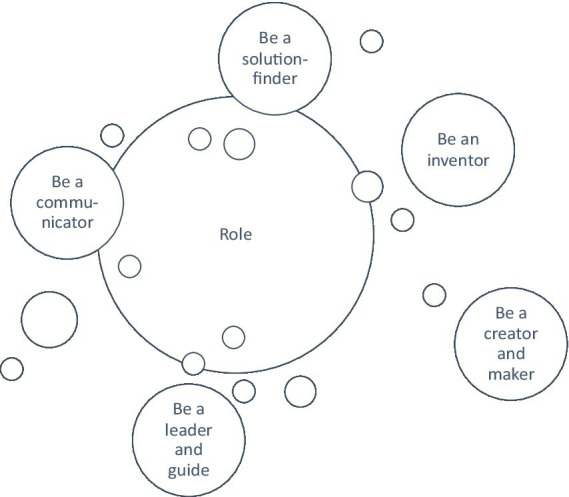
Role.

All of the roles which are being mentioned are somehow experienced as conflictual in a way that managers feel that they are needed, but at the same time emphasize that they are not lived as much as they should be lived within the organization. Some managers emphasize that there is a lack in important roles being taken on within the organization (e.g., P1, P4, P14, P15, P16). All of the managers highlight that they need to take on new roles which relate to the 4IR in a way that managers become communicators to inform employees, that they become solutions-finders, inventors, leaders and guides, as well as creators and makers who communicate the latest developments while finding solutions for the context-specific problem, being inventors to develop and apply new ideas. All of these roles are positive roles which are needed from the perspective of the managers to drive a constructive and successful 4IR (Figure 5).

### Task

Tasks are usually the building blocks of work and they support employees and the organization to overcome boundaries and do the work. Managers within the organization emphasize that they are very busy carrying out their tasks on a daily basis and that all employees should act responsibly and task driven. The managers emphasize that there are tasks that need to be applied on four levels: on societal levels, organizational levels, inter- (managers, customers and employees) and intrapersonal levels (individual tasks). The aspect of transformation is emphasized, expressing that besides the main tasks of the organization to develop, produce and sell their goods, there are underlying tasks that need to be taken on to transform the organization further. These tasks are described on different levels.

Managers highlight that the employees and the organization have a task to fulfill and this is to transform the society, have a societal impact, act with corporate social responsibility (P9, P13) and by bringing 4IR and Black Economic Empowerment (BEE; e.g., P1, P3, P5, P9, P12, P13, P16) together to act for the good of citizens. Thus, the organization’s task is to serve the people in a broader sense and the society and its transformation for the better. Furthermore, the tasks with regard to the company is to create a positive mindset within the organization which is based on information and direction and which complements people’s ability to function optimally and give their best (P7, P8, P10, P13). Another task on organizational level is to move forward in technological development while transforming the jobs within the organization. However, P9 reflects on the transformation, the potential of the individuals and how to cater for others with different needs:

Coming into the fourth industrial revolution? It is about people. People, getting the right people, getting the right people here in South Africa, getting trained people, getting people who want to go that side. They are, not all of them want to go into information technology. There is a lot of mathematics, is a lot about things, a lot about programming…but to transform you need the right people…

The task on an interpersonal level is to identify people who can support the 4IR. On the one hand, 4IR is about transforming the relationships with customers and on the other hand it is about transforming the knowledge, skills and interactions with employees. The majority of managers mention that the work within the organization can only be done when the relationship with customers is transformed in a way that managers relate to them in a technologically advanced (e.g., P8, P10), but at the same time context-specific way. Therefore, the technology is important, but the personal, human interaction with the customer is most important. Managers highlight strongly that it is human interaction that make the business with customers successful—not necessarily the advanced technology (e.g., P2, P6, P8, P12). P2, for example, states:

A lot of our customers do not want that advanced technology. They want a pump that works. And they want service. The personal relationship is very important. It is about that relationship with the customer, not about the technology. They want a product that can withstand the harsh conditions in Africa.

The technological advances which are primarily created in industrialized countries for the industrialized markets need to be brought together with the customers’ needs within African contexts. One of the main tasks of managers is to address this void of interlinkages through communication and adjustment of the technology and advancement. Another task is to transform employees’ knowledge and skills (e.g., P1, P5, P9, P11, P12, P14, P15). Given the fact that the society does not manage to create individuals who are prepared for the labor market, the organization and particularly their managers need to take on the task to train and educate employees for them to gain skills and be informed. Hence, employees need to be trained on the job and off the job.

Finally, an important task of managers is to transform individually and within themselves. They seek new, future-orientated tasks and meaning in their jobs and in their lives, which enrich their life and work (P1, P4, P8, P9, P10, P11, P14, P16). They feel that it is part of their task to develop and grow within their area of expertise and bring about innovation and change to the company for the company’s growth. P8 points out:

It is about new horizons. We need to broaden our horizon and care for the people, for the communities and our environment.

This manager emphasizes the importance of a broader viewpoint to not only focus on success in the 4IR, but also on focusing on the care for others and the world. The new tasks are therefore not only work-related, but rather capture a broader sense-making, development and growth with concern for the consequences of acting.

**Figure 6:**
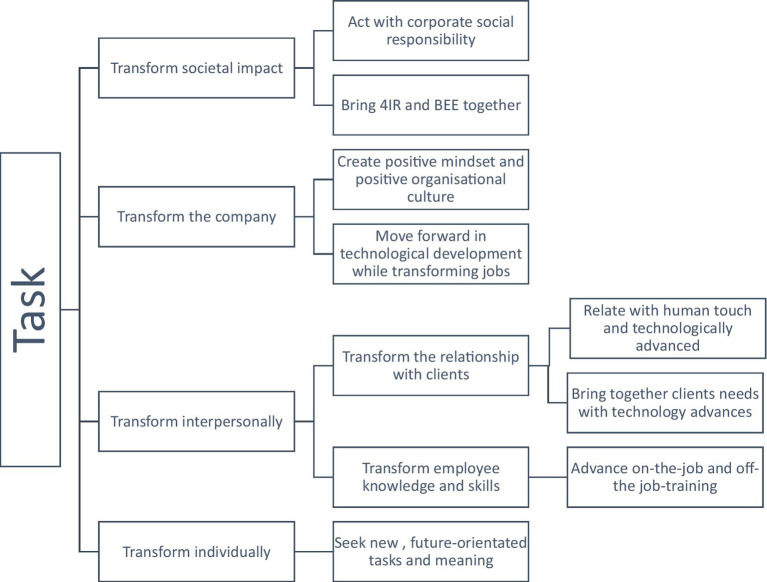
Task.

Focusing on the topic of tasks within the organization (Figure 6), managers see their tasks in the 4IR setting not only being limited to their work context, but also as being broad in a way that these tasks need to take societal, organizational, interpersonal and intrapersonal tasks into consideration to contribute the betterment of the world’s system, to act responsibly, in a social, ecological, human and meaningful way. 4IR tasks reach beyond the classical task on doing the job one is employed to do.

## Discussion

The findings display a wealth of information about the unconscious impact of CIBART within the organization and which might have been underestimated within the organization itself (Tan & Wu, 2017; Durães et al., 2018). Managers within the organization seem to be relatively unaware of the unconscious dynamics within the organization (Steiner and Hall, 2017). Findings show that conflict experiences are strong within the organization, emerging on all levels, as presented by Koortzen and Cilliers (2007), namely on intra-, interpersonal, organizational and societal level. These conflicts interrelate with the occurring identity definitions, and the shifts in boundary, authority, role, and task. Conflicts experienced in the organization seem to evoke in some managers excitement, inventiveness and activity while they evoke in others frustration, anxiety, passiveness and resignation to deal with the transformation. This finding (Mayer, 2008) shows that transformation is an interplay of resignation and resistance on the one hand and assertion, agreement and immersion into the new processes on the other hand. The feeling of not being able to use and go with the advances of the times creates conflict in individuals. Here, conflict experience does not seem to create fight reactions, but rather flight reactions (Koortzen and Oosthuizen, 2012) which lead to a kind of inner flight being expressed in stagnation and resignation. By shedding away the conflict experiences into the unconscious, managers within the organization create a feeling of security (Bion, 1961). This feeling is supported by matching with effective others and by creating (imaginary) subgroups, such as people from the same generation, race, language group, but also employees of advanced knowledge and employees without, employees who see themselves as makers or as passive observers of the shifting powers and technological advances.

The managers are aware that new identities are forming within the transformational era which are higher in complexity and which need to integrate splits and controversial viewpoints in terms of socio-economical, technological and political systems (Hayden and Molenkamp, 2004; Oosthuizen and Mayer, 2019). Findings show further that managers experience boundary shifts which impact on the identity formation and which clusters employees in new segments regarding generations (technology-versatile and non-technology-versatile generations), educational and skill boundaries (highly qualified and lowly/not qualified), geo-economic and political boundaries (global and African-orientated) and emotional (positive and negative emotions) boundaries. The findings support the idea of Heracleous (2004), and James and Huffington (2004) that boundaries shape the organizational structure and creation of flow and subgroup alliances and divides. In this study, boundaries show that some are created based on socio-historical boundaries (e.g., educational divides based on access to educational systems during Apartheid and their Post-Apartheid reflections. Divides often occur between members of generational groups (however, here combined with the ability to adjust to technological advances), some might be specific to the organization being based in Germany (headquarters) and South Africa (subsidiary), always having to negotiate the boundary between global, German and African approaches and decision-making. The emotional boundaries create the space between the individual and other employees, as described by Cilliers and Koortzen (2005), dividing the employees into generally positive and negative thinkers regarding 4IR and the organization’s transformation.

The study shows that managers ascribe the new authority to technology on internal and external levels, defining themselves and the abilities of others in constant comparison with technological knowhow. Technology guides the setting of priorities, the judgment of abilities of self and others (Beck and Visholm, 2014). It appears as this “technological authority” is given from management, but also from the organizational group itself and from the lower level since employees on all levels of the organization seem to believe that technology is the set standard in the present tense. In terms of the roles defined by managers, it appears that ascribed roles seem to be mainly self-ascribed and appear to primarily refer to existential roles, rather than to be normative or phenomenal roles (e.g., Cytrynbaum and Noumair, 2004). Most of the roles (solution-finder, inventor, maker/creator, communicator and leader/guide) are individually defined roles, not roles that standardize the organizational roles, but rather work with the individual and creative impact of the managers.

Finally, managers relate to task as the building pieces of their work as described by Cilliers and Koortzen (2005), because they use their tasks to transform the organizations toward 4IR, the society, the individual relationships and the individuals themselves for the improvement on all levels of the organization. The tasks referred to by managers in this organization also request to understand other aspects within the society and the organization (e.g., BEE, job transformation, corporate social responsibility, customer-relationships and individual tasks and meaning creation). For that reason most of the tasks seem to be essential while they also have an auxiliary function (Obholzer and Roberts, 1994; Cytrynbaum and Noumair, 2004). Hardly any tasks which are described seem to be off-task or anti-task.

## Conclusion and recommendations: Managing leadership and change

In conclusion, findings show that the new technological advances and the transformation into the 4IR make the unconscious dynamics within the organization play out at a new level. Through the changes and the high demand of a properly working infrastructure on societal level, conflict and infrastructural problems in the society play out directly in the organization and create conflicting situations which creates frustration and hampers organizational development. On an organizational level, a lack of trust in the mother organization and decision-making structures becomes relevant and a territorial and competence struggle emerges between the subsidiary and its headquarters. Further, interpersonal and intrapersonal interactions occur based on the experienced split of people with regard to their knowledge, ability to develop and manage the change while developing their interpersonal cooperation and their selves. Conflict occurs in particular where individuals feel that parts of the organization will be left behind because they are not prepared for the advancement. This fear which goes with the split of the advancing and the not advancing employees does not only create conflict inter-, but also intrapersonally.

In terms of identity, findings show that a far more complex understanding of identity (organizational, managerial, individual identity) is needed in the 4IR to create synergies, connect and behave in an inclusive and integrative manner to play at the top of the industry and be successful. Therefore, identities need to be boundary-spanning and reach from local to global identities to create holistic and functioning identity concepts. It further seems to be that boundaries are in particular drawn with regard to the split regarding technological know-how. These boundaries play out along generational, cultural, racial, language, educational and emotional lines on the individual and organizational levels and become more distinct. The unconscious boundaries clearly indicate who is part of the 4IR and who is not.

Findings show a strong shift in terms of authority which is turning away from authority of individuals and humans toward technology-based authority. This shift in authority goes along with an experienced loss of responsibility, control and insight. Managers seem to adhere to the idea that they are no longer in charge of the organization, that they have hardly any influence and say, but that they rather try to keep up with change in which they have no impact. This development seems to be very dangerous because it seems that the new authority is the self-sufficient and self-ruling technology, not the human mind.

In the 4IR process, managerial roles which are seen as key playing roles include the role of the communicator (between humans and machines), the solutions-finder, inventor, leader and guide, creator and maker which all use a mixture of global and local knowledge for the success of the organization. Managers rather become mediators than drivers and seem to fear this shift in roles. Finally, managers see new tasks which are not limited to their organization, but to the broader picture. Tasks of each and every individual are viewed as reaching beyond the conventional organizational task and need to consider the global footprint.

It is recommended that future research takes the unconscious dynamics within organizations increasingly into account to explore the challenges of these changes, its advantages as well as its dangers. The authority and role of technology given to and taken by technological advantages need to be further explored and understood with regard to other concepts, such as responsibility, impact, and complexity. It needs to be explored who the new leaders of organizations are who are prepared to deal with the underlying unconscious dynamics and drive employees through these shifts and changes with a positive attitude, an active and responsible mindset which will work out for the better not only for the self, the organization and the country, but for the world.

Based on research that goes beyond the obvious, organizational practice needs to be developed. Employees need to be trained to become aware of the ongoing 4IR processes, of their own possibility of impact in these changes, and of the need to become an active and influential key player and element within the unconscious systems psychodynamics. Employees need to be trained to understand how to deal with the complex changes and how CIBART plays out on individual, organizational, societal and global levels to find their own conscious viewpoint and define their standing within the process to actively impact on the developments where needed.

## Data availability statement

The raw data supporting the conclusions of this article will be made available by the authors, without undue reservation.

## Ethics statement

The studies involving human participants were reviewed and approved by University of Johannesburg. The patients/participants provided their written informed consent to participate in this study.

## Author contributions

All authors listed have made a substantial, direct, and intellectual contribution to the work and approved it for publication.

## Conflict of interest

The authors declare that the research was conducted in the absence of any commercial or financial relationships that could be construed as a potential conflict of interest.

## Publisher’s note

All claims expressed in this article are solely those of the authors and do not necessarily represent those of their affiliated organizations, or those of the publisher, the editors and the reviewers. Any product that may be evaluated in this article, or claim that may be made by its manufacturer, is not guaranteed or endorsed by the publisher.
